# Discovery of Cinnamic Acid Derivatives as Potent Anti-*H. pylori* Agents

**DOI:** 10.3390/molecules29194548

**Published:** 2024-09-25

**Authors:** Yonglian Li, Kun Zhao, Zhidi Wu, Yujun Zheng, Jialin Yu, Sikun Wu, Vincent Kam Wai Wong, Min Chen, Wenfeng Liu, Suqing Zhao

**Affiliations:** 1 Department of Pharmaceutical Engineering, School of Biomedical and Pharmaceutical Sciences, Guangdong University of Technology, Guangzhou 510006, China; liyli85@126.com (Y.L.); a1002797984@gmail.com (K.Z.); wuzhidixmt@163.com (Z.W.); 13076863429@163.com (Y.Z.); 2112312001@mail2.gdut.edu.cn (S.W.); 2 School of Eco-Environment Technology, Guangdong Industry Polytechnic University, Guangzhou 510300, China; 3 School of Pharmacy and Food Engineering, Wuyi University, Jiangmen 529020, China; yjl13794146710@126.com (J.Y.); cmin0501@outlook.com (M.C.); 4 Neher’s Biophysics Laboratory for Innovative Drug Discovery, State Key Laboratory of Quality Research in Chinese Medicine, Macau University of Science and Technology, Macau 999078, China; bowaiwong@gmail.com

**Keywords:** cinnamic acid derivatives, *Helicobacter pylori*, in vitro inhibition, mechanism

## Abstract

Antibiotics are currently used for the treatment of *Helicobacter pylori* (*H. pylori*), which is confirmed to be the major cause of gastric disorders. However, the long-term consumption of antibiotics has already caused antibiotic resistance and side effects in vivo. Therefore, there is an emerging need for searching for safe and effective anti-*H. pylori* agents. Inspired by the excellent bioactivities of cinnamic acid, a series of cinnamic acid derivatives (compounds **1**–**30**) were synthesized and determined for *H. pylori* inhibition. The initial screening revealed that compound **23**, a 2,4-dinitro cinnamic acid derivative containing 4-methoxyphenol, showed excellent *H. pylori* inhibition with an MIC value of 4 μM. Further studies indicated that compound **23** showed anti-bacterial activity and had a bactericidal effect on *H. pylori* due to the destruction of the bacterial structure. Molecular docking analysis revealed that the 2,4-dinitro groups in cinnamic acid moiety formed hydrogen bonding with amino acid residues in an active pocket of *H. pylori* protein. Interestingly, the ester moiety fitted into the hydrophobic pocket, attaining additional stability to compound **23**. Above all, the present study reveals that compound **23** could be considered a promising anti-*H. pylori* agent to treat *H. pylori* causing gastritis.

## 1. Introduction

*Helicobacter pylori* (*H. pylori*), colonized in the gastric mucosa of humans, is a Gram-negative bacillary spiral-shaped bacterium [1]. It is revealed that *H. pylori* was closely associated with a series of stomach-related diseases, such as chronic gastritis and gastric ulcer [2,3]. Notably, *H. pylori* has been officially categorized as a class-I carcinogen, which is associated with gastric cancer risk [4]. Recent studies demonstrate that eradication of *H. pylori* could reduce the risk of gastric cancer and improve the healing of peptic ulcers [5,6]. Currently, non-antibiotic drugs combined with one/two antibiotics and antibiotic-based therapies are used for the contemporary therapeutic strategies to treat *H. pylori* infection [7]. In addition, antibiotic-based therapies are primarily used to treat *H. pylori* infection, including standard triple therapy and quadruple therapy [8]. However, the increasing antibiotic resistance in *H. pylori* is receiving considerable attention in antibiotic-based therapy [9]. Additionally, the overuse of antibiotics for *H. pylori* infection has caused side effects, such as nausea, constipation, diarrhea and appetite disorder [10]. These disadvantages are the key challenges for treating *H. pylori* infection. Herein, developing novel strategies are urgently needed to prevent and treat *H. pylori* infection.

**Figure 1:**
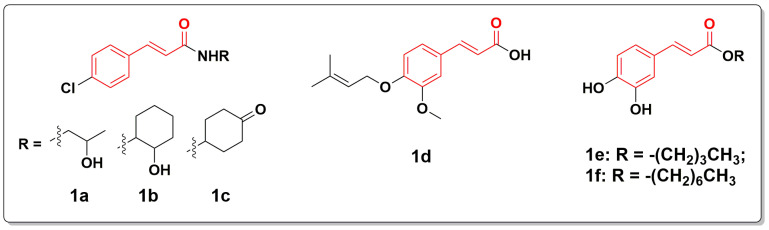
The chemical structures of reported cinnamic acid derivatives (**1a**–**1f**).

Since antibiotics are becoming less effective, natural phytochemicals that have antimicrobial activity are attractive candidates [11,12,13]. Several natural phytochemicals are reported to exhibit fewer side effects, low toxicity and good anti-bacterial activity, indicating that they can serve as potent anti-bacterial drugs [14]. Therefore, discovering anti-bacterial drugs from nature-based compounds is an important strategy for the treatment of *H. pylori* infection [15]. Cinnamon, obtained from the inner bark of several trees from the genus *Cinnamomum*, is widely used as a tropical Asian spice in daily diet [16]. It possesses anti-bacterial, anti-diabetic, anti-inflammatory and anticancer activities [17,18,19,20]. Cinnamic acid, a secondary metabolite of Cinnamon, is responsible for its anti-bacterial activity [21]. Furthermore, it has low toxicity [22,23]. In recent years, it has been recognized that cinnamic acid and its derivatives have significantly contributed to anti-*H. pylori* infection [24,25,26]. It is reported that a series of *N*-substituted cinnamamides were synthesized and screened for anti-*H. pylori* activity. The results showed that three 4-chloro derivatives exhibited excellent anti-*H. pylori* activities with minimum inhibitory concentration (MIC) values in the range of 7.5–10 μg/mL, suggesting that they were the most promising compounds to treat *H. pylori* infection. (Figure 1 (**1a**–**1c**)) [24]. Prenyloxy cinnamic acid derivatives, found in *Boronia pinnata* (*Fam. Rutaceae*), were determined for the growth inhibition of *H. pylori*. Furthermore, compound **1d** had significant inhibition against *H. pylori* (MIC value of 1.62 mg/mL) [25]. It is revealed that cinnamic acid derivatives were synthesized and tested for anti-*H. pylori* activity. The obtained MIC values for compounds **1e** and **1f** were 250 μg/mL, which were four times less than that of caffeic acid [26]. Nevertheless, these observations indicate that there was no systemic research in cinnamic acid-based anti-*H. pylori* agents, especially for the anti-*H. pylori* mechanism. Thus, it is strongly needed to develop novel anti-*H. pylori* agents and exploring the mechanism of action.

Aiming to obtain novel cinnamic acid-based compounds with inhibitory effects on *H. pylori*, a series of cinnamic acid derivatives are synthesized and screened for their anti-*H. pylori* activity. Moreover, the MIC/minimum bactericidal concentration (MBC) and kinetic studies of the most potent compound are explored. The present study can accelerate follow-up research on cinnamic acid derivatives as a potential therapy in the treatment of *H. pylori* infection.

## 2. Results and Discussion

### 2.1. Chemistry

It is indicated that cinnamic acid derivatives had an inhibitory effect on *H. pylori* [23]. In addition, phenol esters were confirmed to be potent *H. pylori* inhibitors [27]. Herein, compounds having both these structural features might possess anti-*H. pylori* activity more efficiently than that of cinnamic acid or phenol esters alone. With this hypothesis, cinnamic acid derivatives containing 4-methoxyphenol were designed for synthesis.

**Scheme 1:**
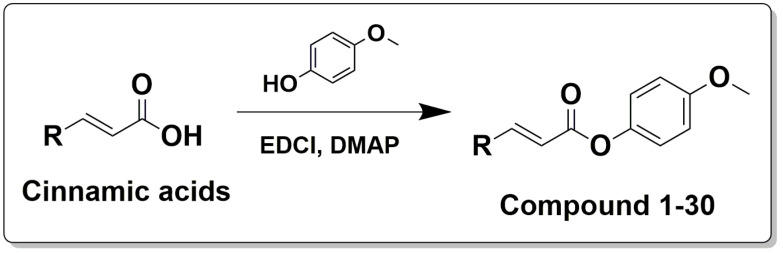
Reagents and conditions: 4-methoxyphenol, EDCI, DMAP, CH_2_Cl_2_, 25 °C, 2 h.

As shown in Scheme 1, various cinnamic acids and 4-methoxyphenol were taken as the starting materials and then esterified into their aralkylacrylate in the presence of 4-dimethylaminopyridine (DMAP) as a catalyst. Firstly, the ester formation was monitored by thin-layer chromatographic (TLC) analysis. Secondly, the chemical structures of compounds **1**–**30** were confirmed by nuclear magnetic resonance (NMR) spectroscopy.

In all ^1^H-NMR spectrums, the olefinic protons appeared as a doublet with coupling constant *J* = 15.7–16.2 Hz. The results confirmed that the double bond in compound **1**–**30** was a trans double bond. In addition, the CH_3_O proton signals were detected as doublets with the range of 3.94–3.81 ppm. In all ^13^C-NMR spectrum, the O=C-O signals were observed between 166.75 and 163.80 ppm. The CH_3_O signals were detected as singlets between 55.64 and 55.61 ppm in the ^13^C-NMR spectrum for all compounds.

### 2.2. Inhibitory Effect of All Compounds against H. pylori

As presented in Table 1, the results indicated that twenty-four compounds that had different *O*-substituted ester moieties exhibited more potent inhibition than that of cinnamic acid at 32 μM (57.30%). The initial screening revealed that seven compounds (compounds **2**, **16**, **19**, **21**, **23**, **24** and **25**) showed the most *H. pylori* inhibitory activity at 32 μM (up to 90% inhibition). Additionally, compounds **2** and **5,** where phenyl was *ortho*-substituted with CH_3_ and OCH_3_, had higher inhibition compared to compounds **3**, **4**, **6** and **7** with CH_3_ and OCH_3_ at *meta* or *para* position, respectively. The inhibitory effect of **8**, **11**, **14**, **17** and **20,** where phenyl was *p*-substituted with F, Cl, Br, CF_3_ and NO_2_, was weaker than that of compounds at *meta* or *para* position. Interestingly, the change in the benzyl group into naphthalene caused a loss of anti-*H. pylori* activity. The compounds (**24**, **25**, **28** and **29**) possessing heterocyclic group showed more potency than that of compounds **26** and **27,** which had benzene rings, indicating that O, S and N atoms in aromatic rings enhanced *H. pylori* inhibition.

According to the MIC values, cinnamic acid alone did not have any effect on *H. pylori* activity. Twenty-nine compounds exhibited significant inhibition with an MIC in the range of 4 μM to 64 μM, indicating that they were more potent than cinnamic acid (MIC > 64 μM). Compound **23** with NO_2_ at *ortho* and *para* position and compound **25** with a quinoline ring exhibited profound inhibition against *H. pylori*. Importantly, compound **25** presented MIC values (8 μM) comparable to metronidazole (8 μM) against *H. pylori*. Compound **23** showed higher *H. pylori* inhibitory activity with a MIC value of 4 μM compared to metronidazole (8 μM). 

Taken together, it is concluded that the increased hydrophobicity of esters might lead to a major structural modification of the in vitro anti-*H. pylori* activity. Importantly, compounds possessing electron-donating groups (CH_3_ and OCH_3_) at *ortho*-position enhanced the anti-*H. pylori* activity. Conversely, compounds possessing electron-withdrawing groups (F, Cl, Br, CF_3_ and NO_2_) at *ortho*-position attenuated the anti-*H. pylori* activity. Considering these results, the promising compound **23** with a MIC value of 4 μM was selected for the experiments.

### 2.3. MIC and MBC

In order to investigate the anti-bacterial activity of compound **23** against *H. pylori*, the microbroth dilution method for the determination of its MIC and MBC was used in the present study. As shown in Table 2, the MIC value of compound **23** against *H. pylori* was 4. The obtained results indicated that the MBC value of compound **23** against *H. pylori* was 8 μM. Importantly, the MBC/MIC ratio of compound **23** was 2, indicating that it showed bacteriostatic and bactericidal activity against *H. pylori*. 

### 2.4. Inhibiting Kinetics and Killing Kinetics of Compound **23** against H. pylori

**Figure 2:**
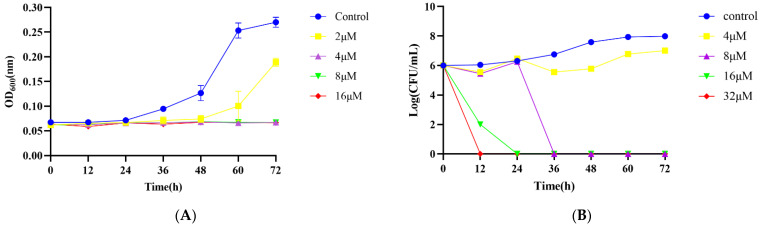
In vitro anti-*H. pylori* activity of compound **23**: (**A**) inhibiting kinetics curves of compound **23** against *H. pylori*; (**B**) killing kinetics curves of compound **23** against *H. pylori*.

As exhibited in Figure 2A,B, the optical density at 600 nm (OD_600_) of *H. pylori* solution was significantly increased after 60 h without drug treatment. Compound **23** dose- and time-dependently inhibited and killed the *H. pylori.* Moreover, compound **23** showed potent in vitro inhibition of *H. pylori* growth at a concentration as low as 2 μM (1/2 MIC). Compound **23** completely killed *H. pylori* at 8–32 μM (2 MIC to 8 MIC) after 12–36 h, which suggested a dramatic decrease in the number of bacteria in comparison to the initial inoculation (Figure 2B). These data revealed that compound **23** showed anti-bacterial activity and had a bactericidal effect on *H. pylori*, indicating that they may serve as potential anti-*H. pylori* agents.

### 2.5. Effect of Compound **23** on the Morphology of H. pylori

**Figure 3:**
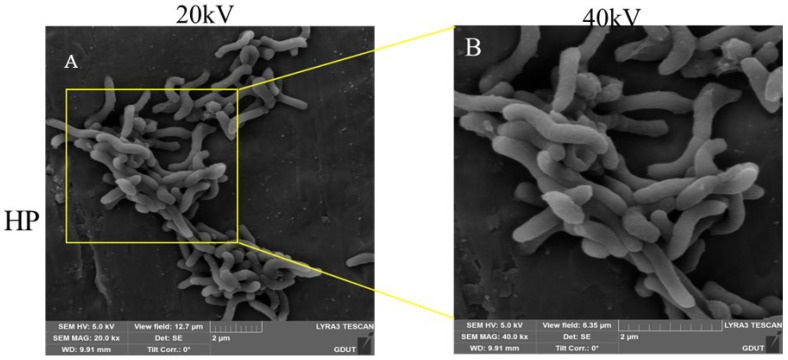

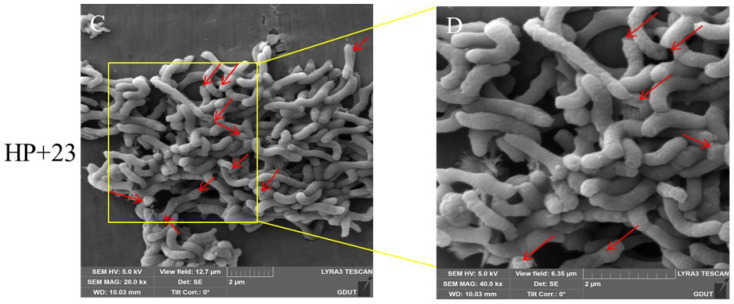
Effect of compound **23** on the biofilm of *H. pylori*. (**A**) SEM images of ATCC SS1 (20 kV); (**B**) SEM images of ATCC SS1 (40 kV); (**C**) SEM images of ATCC SS1 after treated by compound **23** at MIC (20 kV); (**D**) SEM images of ATCC SS1 after treated by compound **23** at MIC (40 kV). Arrows represent morphological changes of *H. pylori* cells on SEM; yellow square represents local site amplification images of **A** and **C**.

To explore the effect of compound **23** at the concentration of MIC on *H. pylori* ultrastructure, the samples were tested via scanning electron microscopy (SEM). As described in Figure 3A,B, the *H. pylori* in the control group showed a smooth, uniform and curved rod-like shape biofilm structure. After treatment with compound **23**, the biofilm structure of *H. pylori* was damaged and shrunk in the compound **23** treated group (Figure 3C,D). They could alter the morphology of *H. pylori*. These results indicated that compound **23** could destroy the structure of *H. pylori* to cause the loss of *H. pylori* activity.

### 2.6. Molecular Docking Analysis

To unravel the molecular interactions of compound **23** with *H. pylori*, molecular docking was used to infer the binding capacity of compound **23** to *H. pylori* (PDB code: 4TSD). It was indicated that HP1029 belonged to the DUF386 family, which was implicated in the bacterial biofilm formation. Additionally, metronidazole, which had an influence on bacterial biofilm formation, has been currently used to treat *H. pylori* infection. Therefore, metronidazole was used as a positive control. The results indicated that docking and fitting (metronidazole and compound **23**) calculated a binding energy value of −7.9 and −5.2 kcal/mol, respectively. The root mean square distance (RMSD) values of metronidazole and compound **23** between the docking pose and the binding configuration in the crystallographic model were 2.120 and 0.625, respectively. It was revealed that compound **23** had a higher affinity for the *H. pylori* protein than that of metronidazole. In addition, the docking program predicted a good pose of metronidazole and compound **23** in the binding pocket. These findings further assisted in screening cinnamic acid derivatives for lead identification and optimization.

**Figure 4:**
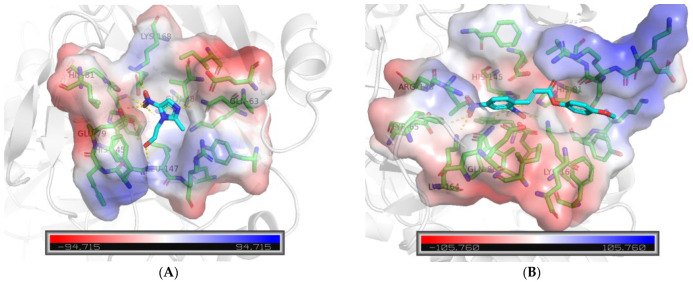
Molecular docking study on the binding of metronidazole (**A**) and compound **23** (**B**) with *H. pylori* as analyzed using Pymol 3.7 software.

As presented in Figure 4A,B, the result indicated that metronidazole and compound **23** were fitted well into the binding pocket of the *H. pylori* protein, respectively. Furthermore, the nitrogen of the nitro group in metronidazole built hydrogen bonds with LYS-168, GLU-79 and HIS-81. The hydroxyl moiety formed hydrogen bonding with HIS145 and LEU-147. The nitrogen of the imidazole ring built hydrogen bonds with GLU-63 and GLN-88. It is demonstrated that the interactions of metronidazole with amino-acid residues could result in the inhibition of the active sites in *H. pylori*. Figure 4B indicated that the nitro group of compound **23** formed hydrogen bonding with LYS-168, HIS-81, HIS145, GLN-88, TYR-65, LYS-164 and ARG-148. Interestingly, the phenyl ring and ester moiety fitted snugly into the hydrophobic pocket, attaining additional stability to compound **23** in the active pocket. Taken together, it is suggested that the good binding affinity of compound **23** with the active site residues would enhance its anti-*H. pylori* activity.

## 3. Materials and Methods

### 3.1. Materials

Cinnamic acid, *p*-methoxyphenol, metronidazole and dimethyl sulfoxide (DMSO) were obtained from Aladin Scientific Corp. (Shanghai, China). Cinnamic acids were synthesized in our laboratory. The melting points of all compounds were measured on a micro melting point apparatus (Shanghai, China). ^1^H and ^13^C NMR spectra of all compounds were measured on a Bruker 500 NMR spectrometer (Bruker, MA, USA). High-resolution EI mass spectra were recorded on a Thermo Scientific liquid chromatography–mass spectrometer (LC-MS, LCQ^TM^, Thermo Scientific, Waltham, MA, USA) with an ESI source. The *H. pylori* ATCC SS1 strain was obtained from Professor Ye Chen’s laboratory at Southern Medical University. Columbia agar base and brain heart infusion (BHI) were acquired from HuanKai Microbial Ltd., (Guangzhou, Guangdong, China). Fetal bovine serum (FBS) was purchased from TianHang Biotech (Hangzhou, Zhejiang, China). Sterile-defibrinated sheep blood was obtained from Ruite Biotechnology Co., Ltd., (Guangzhou, Guangdong, China). Unless otherwise indicated, all solvents, reagents and the other materials were directly used as obtained from the indicated commercial sources without further purification.

### 3.2. General Procedure for the Preparation of Compound **1**–**30**

The procedure for the preparation of compound **1**–**30** is as follows: a mixture of cinnamic acids (1.0 mmol), *p*-methoxyphenol (1.0 mol), 1-ethyl-3-(3-dimethylaminopropyl)-carbodiimide (EDCI, 1.2 mmol)) and DMAP (0.1 mmol) was first dissolved in CH_2_Cl_2_. The reaction was carried out by continuous stirring at room temperature for 1 h. TLC was used to monitor these reactions. After completion of the chemical reaction, the mixture was washed twice with saturated NaHCO_3_ solution and water. Subsequently, the organic phase was collected, dried on anhydrous Na_2_SO_4_ and concentrated under vacuum. Finally, purification through column chromatography afforded the target compound **1**–**30**.

*4-methoxyphenyl cinnamate* (**1**): White solid, 76% yield, mp 125.3–126.0 °C; ^1^H NMR (500 MHz, CDCl_3_) δ 7.87 (d, *J* = 16.0 Hz, 1H, Ph-H), 7.62–7.56 (m, 2H, Ph-H), 7.46–7.40 (m, 3H, 3 × Ph-H), 7.12–7.06 (m, 2H, 2 × Ph-H), 6.96–6.91 (m, 2H, Ph-H and -CH=C-), 6.63 (d, *J* = 16.0 Hz, 1H, -CH=C-), 3.82 (s, 3H, -OCH_3_). ^13^C NMR (126 MHz, CDCl_3_) δ 165.84 (-C=O), 157.26 (Ph-C-OCH_3_), 146.43 (-CH=CH), 144.29 (Ph-C-O), 134.24 (Ph-C-CH), 130.69 (Ph-CH), 129.02 (Ph-CH), 128.31 (Ph-CH), 122.41 (Ph-CH), 117.38 (=CH-C=O), 114.51 (Ph-CH), 55.63 (-O-CH_3_). HRMS *m*/*z*: 255.0943 [M + H]^+^, found 255.0957.

*4-methoxyphenyl (E)-3-(o-tolyl)acrylate* (**2**): White solid, 85% yield, mp 108.9–109.2 °C; ^1^H NMR (500 MHz, CDCl_3_) δ 8.18 (d, *J* = 15.9 Hz, 1H, Ph-H), 7.66 (dd, *J* = 7.7, 1.4 Hz, 1H, Ph-H), 7.34 (td, *J* = 7.4, 1.4 Hz, 1H, Ph-H), 7.27 (dd, *J* = 7.6, 3.7 Hz, 2H, 2 × Ph-H), 7.15–6.93 (m, 4H, 3 × Ph-H and -CH=C-), 6.58 (d, *J* = 15.9 Hz, 1H, -CH=C-), 3.85 (s, 3H, -OCH_3_), 2.51 (s, 3H, -CH_3_). ^13^C NMR (126 MHz, CDCl_3_) δ 165.89 (-C=O), 157.25 (Ph-C-OCH_3_), 144.33 (-CH=CH), 144.08 (Ph-C-O), 137.96 (Ph-C-CH_3_), 133.19 (Ph-C-CH), 130.93 (Ph-CH), 130.42 (Ph-CH), 126.59 (Ph-CH), 126.46 (Ph-CH), 122.42 (Ph-CH), 118.36 (=CH-C=O), 114.48 (Ph-CH), 55.62 (-O-CH_3_), 19.87 (-CH_3_). HRMS *m*/*z*: 269.1099 [M + H]^+^, found 269.1107.

*4-methoxyphenyl (E)-3-(m-tolyl)acrylate* (**3**): White solid, 94% yield, mp 144.0–145.1 °C; ^1^H NMR (500 MHz, CDCl_3_) δ 7.86 (d, *J* = 15.9 Hz, 1H, Ph-H), 7.42 (dd, *J* = 6.6, 1.5 Hz, 2H, 2 × Ph-H), 7.37–7.31 (m, 1H, Ph-H), 7.27 (d, *J* = 7.6 Hz, 1H, Ph-H), 7.15–7.10 (m, 2H, 2 × Ph-H), 6.97–6.93 (m, 2H, Ph-H and -CH=C), 6.64 (d, *J* = 16.0 Hz, 1H, -CH=C), 3.84 (s, 3H, -OCH_3_), 2.42 (s, 3H, -CH_3_). ^13^C NMR (126 MHz, CDCl_3_) δ 165.90 (-C=O), 157.24 (Ph-C-OCH_3_), 146.62 (Ph-C-O), 144.32 (-CH=CH), 138.69 (Ph-C-CH_3_), 134.19 (Ph-C-CH), 131.53 (Ph-CH), 128.98 (Ph-CH), 128.91 (Ph-CH), 125.50 (Ph-CH), 121.43 (Ph-CH), 117.12 (=CH-C=O), 114.49 (Ph-CH), 55.62 (-O-CH_3_), 21.36 (-CH_3_). HRMS *m*/*z*: 269.1099 [M + H]^+^, found 269.1107.

*4-methoxyphenyl (E)-3-(p-tolyl)acrylate* (**4**): White solid, 78% yield, mp 156.2–157.0 °C; ^1^H NMR (500 MHz, CDCl_3_) δ 7.84 (d, *J* = 16.0 Hz, 1H, Ph-H), 7.49 (d, *J* = 8.1 Hz, 2H, 2 × Ph-H), 7.23 (d, *J* = 8.0 Hz, 2H, 2 × Ph-H), 7.12–7.06 (m, 2H, 2 × Ph-H), 6.96–6.88 (m, 2H, Ph-H and -CH=C-), 6.59 (d, *J* = 16.0 Hz, 1H, -CH=C-), 3.82 (s, 3H, -OCH_3_), 2.40 (s, 3H, -CH_3_). ^13^C NMR (126 MHz, CDCl_3_) δ 166.05 (-C=O), 157.22 (Ph-C-OCH_3_), 146.47 (Ph-C-O), 144.35 (-CH=CH), 141.21 (Ph-C-CH_3_), 131.52 (Ph-C-CH), 129.76 (Ph-CH), 128.33 (=CH-C=O), 122.44 (Ph-CH), 116.23 (Ph-CH), 114.49 (Ph-CH), 55.62 (-O-CH_3_), 21.56 (-CH_3_). HRMS *m*/*z*: 269.1099 [M + H]^+^, found 269.1104.

*4-methoxyphenyl (E)-3-(2-methoxyphenyl)acrylate* (**5**): White solid, 82% yield, mp 114.7–115.2 °C; ^1^H NMR (500 MHz, CDCl_3_) δ 8.19 (d, *J* = 16.1 Hz, 1H, Ph-H), 7.59 (dd, *J* = 7.8, 1.7 Hz, 1H, Ph-H), 7.41 (ddd, *J* = 8.5, 7.4, 1.7 Hz, 1H, Ph-H), 7.15–7.09 (m, 2H, 2 × Ph-H), 7.02 (t, *J* = 7.5 Hz, 1H, Ph-H), 6.99–6.92 (m, 3H, 2 × Ph-H and -CH=C-), 6.75 (d, *J* = 16.1 Hz, 1H, -CH=C-), 3.94 (s, 3H, -OCH_3_), 3.84 (s, 3H, -OCH_3_). ^13^C NMR (126 MHz, CDCl_3_) δ 166.36 (-C=O), 158.57 (Ph-C-OCH_3_), 157.16 (Ph-C-OCH_3_), 144.44 (-CH=CH), 141.92 (Ph-C-O), 131.90 (Ph-C-CH), 129.33 (Ph-CH), 123.93 (Ph-CH), 122.48 (Ph-CH), 120.7 (Ph-CH), 117.89 (=CH-C=O), 114.45 (Ph-CH), 111.22 (Ph-CH), 55.61 (-O-CH_3_), 55.52 (-O-CH_3_). HRMS *m*/*z*: 285.1049 [M + H]^+^, found 285.1057.

*4-methoxyphenyl (E)-3-(3-methoxyphenyl)acrylate* (**6**): White solid, 56% yield, mp 120.5–121.3 °C; ^1^H NMR (500 MHz, CDCl_3_) δ 7.85 (d, *J* = 15.9 Hz, 1H, Ph-H), 7.36 (t, *J* = 7.9 Hz, 1H, Ph-H), 7.23–7.18 (m, 1H, Ph-H), 7.14–7.09 (m, 3H, 3 × Ph-H), 7.00 (dd, *J* = 8.3, 2.5 Hz, 1H, Ph-H), 6.97–6.92 (m, 2H, Ph-H and -CH=C-), 6.64 (d, *J* = 15.9 Hz, 1H, -CH=C-), 3.87 (s, 3H, -OCH_3_), 3.84 (s, 3H, -OCH_3_). ^13^C NMR (126 MHz, CDCl_3_) δ 165.77 (-C=O), 159.96 (Ph-C-OCH_3_), 157.26 (Ph-C-OCH_3_), 146.34 (Ph-C-O), 144.27 (-CH=CH), 135.58 (Ph-C-CH), 130.02 (Ph-CH), 122.40 (Ph-CH), 121.00 (Ph-CH), 117.66 (=CH-C=O), 116.57 (Ph-CH), 114.50 (Ph-CH), 113.10 (Ph-CH), 55.61 (-O-CH_3_), 55.34 (-O-CH_3_). HRMS *m*/*z*: 285.1049 [M + H]^+^, found 285.1055.

*4-methoxyphenyl (E)-3-(4-methoxyphenyl)acrylate* (**7**): White solid, 97% yield, mp 108.3–109.6 °C; ^1^H NMR (500 MHz, CDCl_3_) δ 7.82 (d, *J* = 15.9 Hz, 1H, Ph-H), 7.54 (d, *J* = 8.7 Hz, 2H, 2 × Ph-H), 7.11–7.05 (m, 2H, 2 × Ph-H), 6.92 (dd, *J* = 12.1, 5.3 Hz, 4H, 3 × Ph-H and -CH=C-), 6.49 (d, *J* = 15.9 Hz, 1H, -CH=C-), 3.85 (s, 3H, -OCH_3_), 3.81 (s, 3H, -OCH_3_). ^13^C NMR (126 MHz, CDCl_3_) δ 166.18 (-C=O), 161.71 (Ph-C-OCH_3_), 157.18 (Ph-C-OCH_3_), 146.11 (Ph-C-O), 144.39 (-CH=CH), 130.03 (Ph-C-CH), 126.97 (Ph-CH), 122.46 (Ph-CH), 114.73 (=CH-C=O), 114.46 (Ph-CH), 55.62 (-O-CH_3_), 55.44 (-O-CH_3_). HRMS *m*/*z*: 285.1049 [M + H]^+^, found 285.1057.

*4-methoxyphenyl (E)-3-(2-fluorophenyl)acrylate* (**8**): White solid, 62% yield, mp 140.2–141.2 °C; ^1^H NMR (500 MHz, CDCl_3_) δ 8.01 (d, *J* = 16.2 Hz, 1H, Ph-H), 7.62 (td, *J* = 7.6, 1.7 Hz, 1H, Ph-H), 7.42 (tdd, *J* = 7.4, 5.1, 1.7 Hz, 1H, Ph-H), 7.22 (td, *J* = 7.6, 1.1 Hz, 1H, Ph-H), 7.18–7.10 (m, 3H, 3 × Ph-H), 6.97–6.92 (m, 2H, Ph-H and -CH=C-), 6.76 (d, *J* = 16.2 Hz, 1H, -CH=C-), 3.84 (s, 3H, -OCH_3_). ^13^C NMR (126 MHz, CDCl_3_) δ 165.67 (-C=O), 162.51 (Ph-CF), 160.49 (Ph-C-OCH_3_), 157.28 (Ph-C-O), 144.25 (-CH=CH), 139.02 (d, *J* = 2.6 Hz) (Ph-C-CF), 132.12 (d, *J* = 8.6 Hz) (Ph-C-CH), 129.33 (d, *J* = 2.7 Hz) (Ph-CH), 124.58 (d, *J* = 3.5 Hz) (Ph-CH), 122.33 (d, *J* = 11.4 Hz) (Ph-CH), 120.00 (d, *J* = 7.0 Hz) (Ph-CH), 116.41 (=CH-C=O), 116.24 (Ph-CH), 114.50 (Ph-CH), 55.61 (-O-CH_3_). HRMS *m*/*z*: 273.0849 [M + H]^+^, found 273.0861.

*4-methoxyphenyl (E)-3-(3-fluorophenyl)acrylate* (**9**): White solid, 67% yield, mp 153.9–154.4 °C; ^1^H NMR (500 MHz, CDCl_3_) δ 8.01 (d, *J* = 16.2 Hz, 1H, Ph-H), 7.62 (td, *J* = 7.6, 1.7 Hz, 1H, Ph-H), 7.42 (tdd, *J* = 7.5, 5.1, 1.7 Hz, 1H, Ph-H), 7.22 (td, *J* = 7.7, 1.1 Hz, 1H, Ph-H), 7.19–7.09 (m, 3H, 3 × Ph-H), 6.97–6.92 (m, 2H, Ph-H and -CH=C-), 6.75 (d, *J* = 16.2 Hz, 1H, -CH=C-), 3.84 (s, 3H, -OCH_3_). ^13^C NMR (126 MHz, CDCl_3_) δ 165.66 (-C=O), 162.51 (Ph-C-OCH_3_), 160.49 (Ph-C-O), 157.28 (Ph-CF), 144.25 (-CH=CH), 139.02 (d, *J* = 2.6 Hz) (Ph-C-CF), 132.11 (d, *J* = 8.9 Hz) (Ph-C-CH), 129.32 (d, *J* = 2.7 Hz) (Ph-CH), 124.57 (d, *J* = 3.6 Hz) (Ph-CH), 122.37 (Ph-CH), 120.00 (d, *J* = 7.0 Hz) (Ph-CH), 116.41 (=CH-C=O), 116.24 (Ph-CH), 114.50 (Ph-CH), 55.62 (-O-CH_3_). HRMS *m*/*z*: 273.0849 [M + H]^+^, found 273.0859.

*4-methoxyphenyl (E)-3-(4-fluorophenyl)acrylate* (**10**): White solid, 84% yield, mp 120.5–122.0 °C; ^1^H NMR (500 MHz, CDCl_3_) δ 7.82 (d, *J* = 16.0 Hz, 1H, Ph-H), 7.60–7.55 (m, 2H, 2 × Ph-H), 7.15–7.05 (m, 4H, 4×Ph-H), 6.95–6.90 (m, 2H, Ph-H and -CH=C-), 6.55 (d, *J* = 16.0 Hz, 1H, -CH=C-), 3.82 (s, 3H, -OCH_3_). ^13^C NMR (126 MHz, CDCl_3_) δ 165.75 (-C=O), 165.14 (Ph-C-OCH_3_), 163.14 (Ph-C-O), 157.28 (Ph-CF), 145.11 (Ph-CH), 144.23 (-CH=CH), 130.52 (Ph-C-CF), 130.47 (Ph-C-CH), 130.26 (Ph-CH), 130.21 (Ph-CH), 122.38 (Ph-CH), 117.10 (=CH-C=O), 116.29 (Ph-CH), 116.12 (Ph-CH), 116.03 (Ph-CH), 114.79 (Ph-CH), 114.51 (Ph-CH), 55.77, 55.62 (-O-CH_3_). HRMS *m*/*z*: 273.0849 [M + H]^+^, found 273.0859.

*4-methoxyphenyl (E)-3-(2-chlorophenyl)acrylate* (**11**): White solid, 74% yield, mp 132.5–132.8 °C; ^1^H NMR (500 MHz, CDCl_3_) δ 8.30 (d, *J* = 16.0 Hz, 1H, Ph-H), 7.71 (dd, *J* = 7.4, 2.0 Hz, 1H, Ph-H), 7.47 (dd, *J* = 7.7, 1.6 Hz, 1H, Ph-H), 7.36 (dtd, *J* = 16.1, 7.3, 1.7 Hz, 2H, 2 × Ph-H), 7.15–7.10 (m, 2H, 2 × Ph-H), 6.98–6.92 (m, 2H, Ph-H and -CH=C-), 6.64 (d, *J* = 16.0 Hz, 1H, -CH=C-), 3.84 (s, 3H, -OCH_3_). ^13^C NMR (126 MHz, CDCl_3_) δ 165.31 (-C=O), 157.30 (Ph-C-OCH_3_), 144.23 (-CH=CH), 142.10 (Ph-C-O), 135.17 (Ph-C-Cl), 132.51 (Ph-C-CH), 131.41 (Ph-CH), 130.30 (Ph-CH), 127.81 (Ph-CH), 127.20 (Ph-CH), 122.37 (Ph-CH), 120.06 (Ph-CH), 114.50 (=CH-C=O), 55.62 (-O-CH_3_). HRMS *m*/*z*: 289.0553 [M + H]^+^, found 289.0560.

*4-methoxyphenyl (E)-3-(3-chlorophenyl)acrylate* (**12**): White solid, 70% yield, mp 135.8–136.2 °C; ^1^H NMR (500 MHz, CDCl_3_) δ 7.81 (d, *J* = 16.0 Hz, 1H, Ph-H), 7.59 (t, *J* = 1.9 Hz, 1H, Ph-H), 7.47 (dt, *J* = 7.4, 1.6 Hz, 1H, Ph-H), 7.43–7.35 (m, 2H, 2 × Ph-H), 7.14–7.09 (m, 2H, 2 × Ph-H), 6.97–6.92 (m, 2H, Ph-H and -CH=C-), 6.64 (d, *J* = 16.0 Hz, 1H, -CH=C-), 3.84 (s, 3H, -OCH_3_). ^13^C NMR (126 MHz, CDCl_3_) δ 165.40 (-C=O), 157.32 (Ph-C-OCH_3_), 144.73 (-CH=CH), 144.17 (Ph-C-O), 136.02 (Ph-C-Cl), 135.04 (Ph-C-CH), 130.52 (Ph-CH), 130.26 (Ph-CH), 128.01 (Ph-CH), 126.45 (Ph-CH), 122.33 (Ph-CH), 118.88 (=CH-C=O), 114.51 (Ph-CH), 55.62 (-O-CH_3_). HRMS *m*/*z*: 289.0553 [M + H]^+^, found 289.0561.

*4-methoxyphenyl (E)-3-(4-chlorophenyl)acrylate* (**13**): White solid, 81% yield, mp 120.1–121.7 °C; ^1^H NMR (500 MHz, CDCl_3_) δ 7.80 (d, *J* = 16.0 Hz, 1H, Ph-H), 7.51 (d, *J* = 8.5 Hz, 2H, 2 × Ph-H), 7.39 (d, *J* = 8.5 Hz, 2H, 2 × Ph-H), 7.11–7.05 (m, 2H, 2 × Ph-H), 6.95–6.89 (m, 2H, Ph-H and -CH=C-), 6.59 (d, *J* = 16.0 Hz, 1H, -CH=C-), 3.81 (s, 3H, -OCH_3_). ^13^C NMR (126 MHz, CDCl_3_) δ 165.63 (-C=O), 157.29 (Ph-C-OCH_3_), 144.96 (-CH=CH), 144.17 (Ph-C-O), 136.62 (Ph-C-Cl), 132.69 (Ph-C-CH), 129.39 (Ph-CH), 122.37 (Ph-CH), 117.92 (=CH-C=O), 114.51 (Ph-CH), 55.63 (-O-CH_3_). HRMS *m*/*z*: 289.0553 [M + H]^+^, found 289.0560.

*4-methoxyphenyl (E)-3-(2-bromophenyl)acrylate* (**14**): White solid, 76% yield, mp 114.0–114.2 °C; ^1^H NMR (500 MHz, CDCl_3_) δ 7.82–7.73 (m, 2H, 2 × Ph-H), 7.60–7.49 (m, 2H, 2 × Ph-H), 7.32 (t, *J* = 7.9 Hz, 1H, 2 × Ph-H), 7.14–7.08 (m, 2H, 2 × Ph-H), 6.98–6.91 (m, 2H, Ph-H and -CH=C-), 6.64 (d, *J* = 16.0 Hz, 1H, -CH=C-), 3.84 (s, 3H, -OCH_3_). ^13^C NMR (126 MHz, CDCl_3_) δ 165.37 (-C=O), 157.32 (Ph-C-OCH_3_), 144.63 (-CH=CH), 144.17 (Ph-C-O), 136.29 (Ph-C-Br), 133.42 (Ph-C-CH), 130.96 (Ph-CH), 130.51 (Ph-CH), 126.87 (Ph-CH), 123.75 (Ph-CH), 122.33 (Ph-CH), 118.90 (=CH-C=O), 114.51 (Ph-CH), 55.63 (-O-CH_3_). HRMS *m*/*z*: 333.0048 [M + H]^+^, found 333.0054.

*4-methoxyphenyl (E)-3-(3-bromophenyl)acrylate* (**15**): White solid, 74% yield, mp 121.6–122.3 °C; ^1^H NMR (500 MHz, CDCl_3_) δ 8.25 (d, *J* = 15.9 Hz, 1H, Ph-H), 7.68 (ddd, *J* = 16.7, 7.9, 1.5 Hz, 2H, 2 × Ph-H), 7.42–7.36 (m, 1H, Ph-H), 7.30 (d, *J* = 1.7 Hz, 1H, Ph-H), 7.27 (d, *J* = 1.6 Hz, 1H, Ph-H), 7.15–7.11 (m, 2H, 2 × Ph-H), 6.97–6.93 (m, 2H, Ph-H and -CH=C-), 6.60 (d, *J* = 15.9 Hz, 1H, -CH=C-), 3.84 (s, 3H, -OCH_3_). ^13^C NMR (126 MHz, CDCl_3_) δ 165.22 (-C=O), 157.30 (Ph-C-OCH_3_), 144.66 (-CH=CH), 144.23 (Ph-C-O), 134.30 (Ph-C-Br), 133.57 (Ph-C-CH), 131.57 (Ph-CH), 127.94 (Ph-CH), 127.83 (Ph-CH), 125.54 (Ph-CH), 122.37 (Ph-CH), 120.24 (Ph-CH), 114.49 (=CH-C=O), 55.63 (-O-CH_3_). HRMS *m*/*z*: 333.0048 [M + H]^+^, found 333.0053.

*4-methoxyphenyl (E)-3-(4-bromophenyl)acrylate* (**16**): White solid, 72% yield, mp 148.7–149.5 °C; ^1^H NMR (500 MHz, CDCl_3_) δ 7.81 (d, *J* = 16.0 Hz, 1H, Ph-H), 7.60–7.56 (m, 2H, 2 × Ph-H), 7.49–7.44 (m, 2H, 2 × Ph-H), 7.14–7.07 (m, 2H, 2 × Ph-H), 6.98–6.92 (m, 2H, Ph-H and -CH=C-), 6.63 (d, *J* = 16.0 Hz, 1H, -CH=C-), 3.84 (s, 3H, -OCH_3_). ^13^C NMR (126 MHz, CDCl_3_) δ 165.57 (-C=O), 157.30 (Ph-C-OCH_3_), 144.99 (-CH=CH), 144.20 (Ph-C-O), 133.13 (Ph-C-Br), 132.28 (Ph-C-CH), 129.65 (Ph-CH), 125.00 (Ph-CH), 122.35 (Ph-CH), 118.07 (=CH-C=O), 114.51 (Ph-CH), 55.62 (-O-CH_3_). HRMS *m*/*z*: 333.0048 [M + H]^+^, found 333.0051.

*4-methoxyphenyl (E)-3-(2-(trifluoromethyl)phenyl)acrylate* (**17**): White solid, 74% yield, mp 102.6–103.5 °C; ^1^H NMR (500 MHz, CDCl_3_) δ 8.26 (dq, *J* = 15.7, 2.2 Hz, 1H, Ph-H), 7.83–7.74 (m, 2H, 2 × Ph-H), 7.63 (t, *J* = 7.6 Hz, 1H, Ph-H), 7.54 (t, *J* = 7.7 Hz, 1H, Ph-H), 7.16–7.11 (m, 2H, 2 × Ph-H), 6.98–6.92 (m, 2H, Ph-H and -CH=C-), 6.62 (d, *J* = 15.7 Hz, 1H, -CH=C-), 3.84 (s, 3H, -OCH_3_). ^13^C NMR (126 MHz, CDCl_3_) δ 164.90 (-C=O), 157.34 (Ph-C-OCH_3_), 144.17 (-CH=CH), 141.77 (Ph-C-O), 133.14 (Ph-C-CH), 132.21 (Ph-CH), 129.94 (Ph-CH), 129.15 (Ph-CH), 128.90 (Ph-CH), 128.04 (Ph-CH), 126.28 (d, *J* = 5.5 Hz) (Ph-C-CF_3_), 125.00 (Ph-CH), 122.82 (Ph-CF_3_), 122.33 (Ph-CH), 121.77 (Ph-CH), 114.49 (=CH-C=O), 55.62 (-O-CH_3_). HRMS *m*/*z*: 323.0817 [M + H]^+^, found 323.0824.

*4-methoxyphenyl (E)-3-(3-(trifluoromethyl)phenyl)acrylate* (**18**): White solid, 82% yield, mp 110.3–111.9 °C; ^1^H NMR (500 MHz, CDCl_3_) δ 7.89 (d, *J* = 16.0 Hz, 1H, Ph-H), 7.85 (s, 1H, Ph-H), 7.77 (d, *J* = 7.8 Hz, 1H, Ph-H), 7.70 (d, *J* = 7.8 Hz, 1H, Ph-H), 7.58 (t, *J* = 7.8 Hz, 1H, Ph-H), 7.14–7.10 (m, 2H, 2× Ph-H), 6.97–6.92 (m, 2H, Ph-H and -CH=C-), 6.72 (d, *J* = 16.0 Hz, 1H, -CH=C-), 3.84 (s, 3H, -OCH_3_). ^13^C NMR (126 MHz, CDCl_3_) δ 165.28 (-C=O), 157.35 (Ph-C-OCH_3_), 144.50 (-CH=CH), 144.14 (Ph-C-O), 135.00 (Ph-C-CH), 131.69 (Ph-CH), 131.43 (Ph-CH), 131.25 (Ph-CH), 129.59 (Ph-CH), 127.02 (d, *J* = 3.5 Hz) (Ph-C-CF_3_), 124.82 (d, *J* = 3.9 Hz) (Ph-C-CF_3_), 122.69 (Ph-CF_3_), 122.31 (Ph-CH), 119.40 (Ph-CH), 114.53 (=CH-C=O), 55.61 (-O-CH_3_). HRMS *m*/*z*: 323.0817 [M + H]^+^, found 323.0820.

*4-methoxyphenyl (E)-3-(4-(trifluoromethyl)phenyl)acrylate* (**19**): White solid, 79% yield, mp 131.7–132.1 °C; ^1^H NMR (500 MHz, CDCl_3_) δ 7.87 (d, *J* = 16.0 Hz, 1H, Ph-H), 7.68 (s, 4H, 4×Ph-H), 7.14–7.05 (m, 2H, 2 × Ph-H), 6.95–6.89 (m, 2H, Ph-H and -CH=C-), 6.70 (d, *J* = 16.0 Hz, 1H, -CH=C-), 3.82 (s, 3H, -OCH_3_). ^13^C NMR (126 MHz, CDCl_3_) δ 165.29 (-C=O), 157.38 (Ph-C-OCH_3_), 144.47 (-CH=CH), 144.12 (Ph-C-O), 137.56 (Ph-C-CH), 132.22 (Ph-CH), 131.96 (Ph-CH), 128.41 (Ph-CH), 125.98 (Ph-CH), 124.89 (Ph-C-CF_3_), 122.72 (Ph-CF_3_), 122.31 (Ph-CH), 119.98 (Ph-CH), 116.02 (=CH-C=O), 114.79 (Ph-CH), 114.54 (Ph-CH), 55.62 (-O-CH_3_). HRMS *m*/*z*: 323.0817 [M + H]^+^, found 323.0823.

*4-methoxyphenyl (E)-3-(2-nitrophenyl)acrylate* (**20**): White solid, 82% yield, mp 11.9–112.4 °C; ^1^H NMR (500 MHz, CDCl_3_) δ 7.88 (d, *J* = 16.0 Hz, 1H, Ph-H), 7.63–7.57 (m, 2H, 2 × Ph-H), 7.44 (dd, *J* = 6.5, 3.4 Hz, 3H, 3 × Ph-H), 7.22 (d, *J* = 8.4 Hz, 2H, 2 × Ph-H), 7.07 (d, *J* = 8.4 Hz, 2H, Ph-H and -CH=C-), 6.64 (d, *J* = 16.0 Hz, 1H, -CH=C-), 2.37 (s, 3H, -OCH_3_). ^13^C NMR (126 MHz, CDCl_3_) δ 164.61 (-C=O), 157.37 (Ph-C-OCH_3_), 148.34 (Ph-C-O), 144.08 (-CH=CH), 141.71 (Ph-C-NO_2_), 133.71 (Ph-C-CH), 130.64 (Ph-CH), 130.44 (Ph-CH), 129.30 (Ph-CH), 125.06 (Ph-CH), 122.48 (Ph-CH), 122.32 (Ph-CH), 114.51 (=CH-C=O), 55.63 (-O-CH_3_). HRMS *m*/*z*: 300.0794 [M + H]^+^, found 300.0799.

*4-methoxyphenyl (E)-3-(3-nitrophenyl)acrylate* (**21**): White solid, 77% yield, mp 120.4–121.5 °C; ^1^H NMR (500 MHz, CDCl_3_) δ 8.43 (t, *J* = 1.7 Hz, 1H, Ph-H), 8.26 (dd, *J* = 8.2, 2.0 Hz, 1H, Ph-H), 7.88 (dd, *J* = 12.0, 3.9 Hz, 2H, 2 × Ph-H), 7.61 (t, *J* = 8.0 Hz, 1H, Ph-H), 7.13–7.04 (m, 2H, 2 × Ph-H), 6.96–6.89 (m, 2H, Ph-H and -CH=C-), 6.77–6.71 (m, 1H, -CH=C-), 3.81 (s, 3H, -OCH_3_). ^13^C NMR (126 MHz, CDCl_3_) δ 165.00 (-C=O), 157.40 (Ph-C-OCH_3_), 148.73 (Ph-C-O), 144.05 (-CH=CH), 143.42 (Ph-C-NO_2_), 135.92 (Ph-C-CH), 133.82 (Ph-CH), 130.12 (Ph-CH), 124.88 (Ph-CH), 122.64 (Ph-CH), 122.26 (Ph-CH), 120.61 (Ph-CH), 114.54 (=CH-C=O), 55.63 (-O-CH_3_). HRMS *m*/*z*: 300.0794 [M + H]^+^, found 300.0801.

*4-methoxyphenyl (E)-3-(4-nitrophenyl)acrylate* (**22**): White solid, 83% yield, mp 156.0–156.4 °C; ^1^H NMR (500 MHz, CDCl_3_) δ 7.88 (d, *J* = 16.0 Hz, 1H, Ph-H), 7.63–7.57 (m, 2H, 2 × Ph-H), 7.44 (dd, *J* = 6.5, 3.4 Hz, 3H, 3 × Ph-H), 7.22 (d, *J* = 8.4 Hz, 2H, 2 × Ph-H), 7.07 (d, *J* = 8.4 Hz, 2H, Ph-H and -CH=C-), 6.64 (d, *J* = 16.0 Hz, 1H, -CH=C-), 2.37 (s, 3H, -OCH_3_). ^13^C NMR (126 MHz, CDCl_3_) δ 164.90 (-C=O), 157.44 (Ph-C-OCH_3_), 148.70 (Ph-C-O), 144.02 (-CH=CH), 143.33 (Ph-C-NO_2_), 140.25 (Ph-C-CH), 128.88 (Ph-CH), 124.28 (Ph-CH), 122.24 (Ph-CH), 121.72 (Ph-CH), 114.56 (=CH-C=O), 55.63 (-O-CH_3_). HRMS *m*/*z*: 300.0794 [M + H]^+^, found 300.0807.

*4-methoxyphenyl (E)-3-(2,4-dinitrophenyl)acrylate* (**23**): White solid, 50% yield, mp 114.6–115.2 °C; ^1^H NMR (500 MHz, CDCl_3_) δ 8.94 (d, *J* = 2.3 Hz, 1H, Ph-H), 8.54 (dd, *J* = 8.6, 2.3 Hz, 1H, Ph-H), 8.31 (d, *J* = 15.8 Hz, 1H, Ph-H), 7.93 (d, *J* = 8.5 Hz, 1H, Ph-H), 7.14–7.09 (m, 2H, 2 × Ph-H), 6.96–6.92 (m, 2H, Ph-H and -CH=C-), 6.67 (d, *J* = 15.8 Hz, 1H, -CH=C-), 3.84 (s, 3H, -OCH_3_). ^13^C NMR (126 MHz, CDCl_3_) δ 163.80 (-C=O), 157.53 (Ph-C-OCH_3_), 148.21 (Ph-C-O), 148.14 (Ph-C-NO_2_), 143.85 (Ph-C-NO_2_), 139.38 (-CH=CH), 136.22 (Ph-C-CH), 131.53 (Ph-CH), 127.81 (Ph-CH), 125.72 (Ph-CH), 122.15 (Ph-CH), 120.68 (Ph-CH), 114.55 (=CH-C=O), 55.64 (-O-CH_3_). HRMS *m*/*z*: 345.0645 [M + H]^+^, found 345.0655.

*4-methoxyphenyl (E)-3-(pyridin-3-yl)acrylate* (**24**): White solid, 64% yield, mp 107.8–108.3 °C; ^1^H NMR (500 MHz, CDCl_3_) δ 8.72–8.69 (m, 2H, 2 × Ph-H), 7.78 (d, *J* = 16.0 Hz, 2H, 2 × Ph-H), 7.45–7.41 (m, 2H, 2 × Pyridine-H), 7.14–7.08 (m, 2H, 2 × Pyridine-H), 6.97–6.91 (m, 1H, -CH=C-), 6.80 (d, *J* = 16.1 Hz, 1H, -CH=C-), 3.83 (s, 3H, -OCH_3_). ^13^C NMR (126 MHz, CDCl_3_) δ 164.87 (-C=O), 157.43 (Ph-C-OCH_3_), 150.71 (Pyridine-CH), 144.01 (Ph-C-O), 143.40 (-CH=CH), 141.34 (Pyridine-C-CH), 122.24 (Pyridine-CH), 122.09 (Ph-CH), 121.90 (Ph-CH), 114.55 (=CH-C=O), 55.62 (-O-CH_3_). HRMS *m*/*z*: 256.0895 [M + H]^+^, found 256.0902.

*4-methoxyphenyl (E)-3-(quinolin-4-yl)acrylate* (**25**): White solid, 70% yield, mp 119.2–120.5 °C; ^1^H NMR (500 MHz, CDCl_3_) δ 8.99 (d, *J* = 4.5 Hz, 1H, Ph-H), 8.59 (d, *J* = 15.9 Hz, 2H, 2 × Ph-H), 8.22–8.17 (m, 2H, 2 × Quinolin-H), 7.80 (ddd, *J* = 8.5, 6.8, 1.3 Hz, 2H, 2 × Quinolin-H), 7.18–7.13 (m, 2H, 2 × Quinolin-H), 6.99–6.93 (m, 2H, Ph-H and -CH=C-), 6.85 (d, *J* = 15.8 Hz, 1H, -CH=C-), 3.84 (s, 3H, -OCH_3_). ^13^C NMR (126 MHz, CDCl_3_) δ 164.83 (-C=O), 157.45 (Ph-C-OCH_3_), 150.15 (Ph-C-O), 148.73 (Quinolin-CH), 144.07 (-CH=CH), 140.79 (Quinolin-C-CH), 139.62 (Quinolin-C-C), 130.31 (Quinolin-C-C), 129.89 (Quinolin-CH), 127.49 (Quinolin-CH), 125.96 (Quinolin-CH), 123.89 (Quinolin-CH), 123.28 (Quinolin-CH), 122.28 (Ph-CH), 118.30 (Ph-CH), 114.57 (=CH-C=O), 55.63 (-O-CH_3_). HRMS *m*/*z*: 306.1052 [M + H]^+^, found 306.1058.

*4-methoxyphenyl (E)-3-(naphthalen-2-yl)acrylate* (**26**): White solid, 79% yield, mp 126.0–127.3 °C; ^1^H NMR (500 MHz, CDCl_3_) δ 8.74 (d, *J* = 15.7 Hz, 1H, Ph-H), 8.27 (d, *J* = 8.5 Hz, 1H, Nap-H), 8.00–7.90 (m, 2H, 2 × Nap-H), 7.87 (d, *J* = 7.2 Hz, 1H, Nap-H), 7.66–7.52 (m, 3H, 3 × Nap-H), 7.21–7.15 (m, 2H, 2 × Ph-H), 7.01–6.95 (m, 2H, Ph-H and -CH=C-), 6.79–6.72 (m, 1H, -CH=C-), 3.86 (s, 3H, -OCH_3_). ^13^C NMR (126 MHz, CDCl_3_) δ 165.73 (-C=O), 157.31 (Ph-C-OCH_3_), 144.35 (-CH=CH), 143.39 (Ph-C-O), 133.73 (Nap-C-C), 131.53 (Nap-C-C), 131.46 (Nap-C-C), 128.83 (Nap-C-CH), 127.07 (Nap-C-CH), 126.37 (Nap-C-CH), 125.53 (Nap-C-CH), 125.32 (Nap-C-CH), 124.73 (Nap-C-CH), 122.46 (Ph-CH), 119.94 (Ph-CH), 114.54 (=CH-C=O), 55.64 (-O-CH_3_). HRMS *m*/*z*: 305.1099 [M + H]^+^, found 305.1107.

*4-methoxyphenyl (E)-3-(6-methoxynaphthalen-2-yl)acrylate* (**27**): White solid, 81% yield, mp 116.7–117.8 °C; ^1^H NMR (500 MHz, CDCl_3_) δ 8.02 (d, *J* = 15.9 Hz, 1H, Ph-H), 7.95–7.93 (m, 1H, Nap-H), 7.82–7.76 (m, 2H, 2 × Nap-H), 7.72 (dd, *J* = 8.5, 1.8 Hz, 1H, Ph-H), 7.21 (dd, *J* = 8.9, 2.5 Hz, 1H, Ph-H), 7.17 (d, *J* = 2.5 Hz, 1H, Nap-H), 7.15–7.11 (m, 2H, 2 × Nap-H), 6.98–6.93 (m, 2H, Ph-H and -CH=C-), 6.70 (d, *J* = 15.9 Hz, 1H, -CH=C-), 3.97 (s, 3H, -OCH_3_), 3.85 (s, 3H, -OCH_3_). ^13^C NMR (126 MHz, CDCl_3_) δ 166.62 (-C=O), 159.44 (Ph-C-OCH_3_), 157.39 (Ph-C-OCH_3_), 146.69 (Ph-C-O), 144.41 (-CH=CH), 136.34 (Nap-C-C), 130.26 (Nap-C-C), 129.62 (Nap-C-C), 128.91 (Nap-C-CH), 127.63 (Nap-C-CH), 124.20 (Nap-C-CH), 122.44 (Nap-C-CH), 119.59 (Nap-C-CH), 116.24 (=CH-C=O), 114.49 (Ph-CH), 106.01 (Ph-CH), 55.63 (-O-CH_3_), 55.43 (-O-CH_3_). HRMS *m*/*z*: 335.1205 [M + H]^+^, found 335.1212.

*4-methoxyphenyl (E)-3-(thiophen-2-yl)acrylate* (**28**): White solid, 77% yield, mp 100.3–101.4 °C; ^1^H NMR (500 MHz, CDCl_3_) δ 7.87 (d, *J* = 15.9 Hz, 1H, Ph-H), 7.59 (dd, *J* = 2.9, 1.3 Hz, 1H, Thiophen-H), 7.39 (qd, *J* = 5.1, 2.0 Hz, 2H, 2 × Ph-H), 7.13–7.08 (m, 2H, 2 × Thiophen-H), 6.97–6.91 (m, 2H, Ph-H and -CH=C-), 6.47 (d, *J* = 15.9 Hz, 1H, -CH=C-), 3.84 (s, 3H, -OCH_3_). ^13^C NMR (126 MHz, CDCl_3_) δ 166.10 (-C=O), 157.23 (Ph-C-OCH_3_), 144.29 (-CH=CH), 139.82 (Ph-C-O), 137.43 (Thiophen-C-CH), 128.87 (Thiophen-C-CH), 127.21 (Thiophen-C-CH), 125.18 (Thiophen-C-CH), 122.41 (Ph-CH), 116.96 (=CH-C=O), 114.49 (Ph-CH), 55.62 (-O-CH_3_). HRMS *m*/*z*: 261.0507 [M + H]^+^, found 261.0513.

*4-methoxyphenyl (E)-3-(furan-2-yl)acrylate* (**29**): White solid, 72% yield, mp 143.5–144.1 °C; ^1^H NMR (500 MHz, CDCl_3_) δ 7.62 (d, *J* = 15.7 Hz, 1H, Ph-H), 7.55 (d, *J* = 1.8 Hz, 1H, Furan-H), 7.13–7.07 (m, 2H, 2 × Ph-H), 6.96–6.91 (m, 2H, 2 × Furan-H), 6.71 (d, *J* = 3.5 Hz, 1H, -CH=C-), 6.55–6.49 (m, 2H, Ph-H and -CH=C-), 3.83 (s, 3H, -OCH_3_). ^13^C NMR (126 MHz, CDCl_3_) δ 165.9 (-C=O), 157.21 (Ph-C-OCH_3_), 150.80 (Ph-C-O), 144.76 (-CH=CH), 143.98 (Furan-C-CH), 132.52 (Furan-CH), 122.40 (Furan-CH), 115.62 (=CH-C=O), 114.89 (Furan-CH), 114.46 (Ph-CH), 112.49 (Ph-CH), 55.61 (-O-CH_3_). HRMS *m*/*z*: 245.0736 [M + H]^+^, found 245.0741.

*4-methoxyphenyl (E)-3-(3,4,5-trimethoxyphenyl)acrylate* (**30**): White solid, 74% yield, mp 150.7–151.0 °C; ^1^H NMR (500 MHz, CDCl_3_) δ 7.77 (d, *J* = 15.9 Hz, 1H, Ph-H), 7.12–7.04 (m, 2H, 2 × Ph-H), 6.94–6.90 (m, 2H, 2 × Ph-H), 6.81 (s, 2H, Ph-H and -CH=C-), 6.53 (d, *J* = 15.9 Hz, 1H, -CH=C-), 3.90 (d, *J* = 1.8 Hz, 9H, -OCH_3_), 3.81 (s, 3H, -OCH_3_). ^13^C NMR (126 MHz, CDCl_3_) δ 165.81 (-C=O), 157.26 (Ph-C-OCH_3_), 153.50 (Ph-C-OCH_3_), 146.38 (Ph-C-O), 144.26 (-CH=CH), 140.40 (Ph-C-CH), 129.70 (Ph-CH), 122.38 (Ph-CH), 116.56 (=CH-C=O), 114.50 (Ph-CH), 105.41 (Ph-CH), 61.03 (-O-CH_3_), 56.19 (-O-CH_3_), 55.62 (-O-CH_3_). HRMS *m*/*z*: 345.1260 [M + H]^+^, found 345.1267.

### 3.3. Bacterial Culture

*H. pylori* was grown and cultured in BHI containing 10% FBS in the presence of oxygen and 100% humidity conditions at 37 °C. The growth of the *H. pylori* ATCC SS1 strain was evaluated using visual inspection after 3 to 5 days.

### 3.4. Anti-Helicobacter pylori Activity

As described in the previous literature [28], we used the modified broth microdilution assay to determine the MIC and the MBC of the target compounds. For the MIC assay, the bacteria were exposed to the target compounds at different concentrations in culture with BHI containing 10% FBS for 72 h. The MICs of all target compounds were tested based on the lowest concentration of the nine compounds, resulting in *Helicobacter pylori* growth inhibition. For the MBC assay, 100 μL solution that contains the target compounds at 1×, 2×, 4× or 8× MICs was removed from the 96-well plate and cultured in a microaerophilic atmosphere at 37 ℃ for 72 h. The MBC was defined as the concentration that killed 99.9% of the initial bacterial population [29]. 

### 3.5. Inhibiting Kinetics and Killing Kinetics Assays

In order to investigate the effect of compounds **23** and **25** against *H. pylori* ATCC SS1 strain, the inhibiting kinetics and killing kinetics assays were used to evaluate the present study. For inhibiting kinetics assays, the ATCC SS1 strain was first exposed to water, compounds **23** or **25**, at low MICs in BHI containing 10% FBS. Then, the bacteria in different groups were shaken at 150 rpm in a tri-gas incubator. Subsequently, 100 μL solution of each group at different times (0, 12, 24, 36, 48, 72 h) were pipetted for absorbance measurement at 600 nm, respectively. Finally, the inhibiting kinetics curves of water, compounds **23** or **25**, were measured and drawn. For killing kinetics assays, the ATCC SS1 strain was firstly exposed to water, compounds **23** or **25**, at high MICs in BHI containing 10% FBS. Secondly, the bacteria in different groups were shaken at 150rpm in a tri-gas incubator and incubated at different times (0, 12, 24, 36, 48, 72 h). Then, 50 μL solution of each group was pipetted for a series of 10-fold dilutions. Subsequently, 100 μL dilutions from different groups were plated on solid agar for several days to form single colonies. Finally, a counter was used to count the number of colonies in different groups. In addition, the results were expressed as Log (CFU/mL). 

### 3.6. SEM

As described previously, the effect of compounds **23** and **25** on *H. pylori* morphology was examined using SEM [30]. Typically, the *H. pylori* ATCC SS1 strain was first incubated in BHI containing 10% FBS with or without compound **23** at the concentration of MIC. They were cultured in a microaerophilic atmosphere at 37 ℃ for 8h. Secondly, after 8 h, the bacteria, treated with compounds **23** and **25**, were collected from the bacterial suspension by centrifugation and washed twice with PBS. The tested samples were subsequently fixed with 2.5% glutaraldehyde and dehydrated through a graduated ethanol series. Finally, the specimens derived from the collected bacteria were treated by metal spraying and observed using a scanning electron microscope.

### 3.7. Molecular Docking

It was indicated that *Helicobacter pylori* HP1029 (PDB ID: 4TSD) was a homodimer, in which each monomer comprised a molecular core formed by 12 antiparallel β-strands arranged in two β-sheets flanked by helices [31]. Moreover, it belonged to the DUF386 family, which was implicated in the bacterial biofilm formation [32]. Therefore, *Helicobacter pylori* HP1029 (PDB ID: 4TSD) was selected for the molecular docking.

As described previously, AutoDock Vina was used to simulate the interaction between metronidazole or compound **23** with HP1029 protein [33]. Firstly, the ligands (metronidazole and compound **23**) were drawn by the ChemDraw 2014 software and then optimized by AutoDock Vina 1.5 software. The hydrogen atoms were added to metronidazole and compound **23** using Babel mode, and partial charges of metronidazole and compound **23** were generated using MOPAC. After that, the hydrogen bonding energy of metronidazole and compound **23** was minimized by using the Lennard-Jones 6/12 potential mode and optimized with iterative minimization. Secondly, a maximum of 300 iterations was used to optimize the energy gradient, and a total of 200 docking runs were performed in each cycle of the iterative minimization. Before docking, the crystal structure of *Helicobacter pylori* HP1029 (PDB ID: 4TSD) was available from the Protein Data Bank and prepared in AutoDock Vina. After all water molecules were removed, the hydrogen atoms were added to make *Helicobacter pylori* HP1029 protein protonation. The active pocket in *Helicobacter pylori* HP1029 protein was simulated by the automatic mode. In addition, other settings were used in the default settings. After the docking analysis was completed, the files were exported from AutoDock software and drawn using the PyMol software. 

### 3.8. Data and Statistical Analysis

Data were presented as mean ± standard error (SD). The results were analyzed using GraphPad Prism 8.0.2 software (GraphPad Software, San Diego, CA, USA). All the statistical analyses were performed by Student’s *t*-test. *p* < 0.05 exhibited a statistically significant difference.

## 4. Conclusions

In summary, the present study describes our efforts to discover a series of cinnamic acid derivatives as potent anti-*H. pylori* agents. Most of the cinnamic acid derivatives exhibited different antimicrobial potency against *H. pylori* activity. Compound **23** emerged as the most potent anti-*H. pylori* agents among the series. Further studies indicated that compound **23** showed anti-bacterial activity and had a bactericidal effect on *H. pylori* due to the destruction of the bacterial structure. Molecular docking analysis revealed the binding pattern of compound **23** interacting with the amino acid residues in the active site. These findings provided a promising rationale for discovering the novel anti-*H. pylori* agents.

## Figures and Tables

**Table 1:** Inhibitory values for cinnamic acid and its derivatives against *H. pylori*.

Compound	R	Inhibition (%) at 32 μM ^a^	MIC (μM) ^a^
Metronidazole	--	99.92 ± 0.10	8
Cinnamic acid	--	57.30 ± 10.38	>64
**1**	Ph	73.29 ± 1.20	64
**2**	2-CH_3_-Ph	98.16 ± 0.73	32
**3**	3-CH_3_-Ph	76.44 ± 4.57	64
**4**	4-CH_3_-Ph	77.60 ± 0.23	64
**5**	2-CH_3_O-Ph	82.03 ± 4.30	64
**6**	3-CH_3_O-Ph	50.58 ± 3.82	64
**7**	4-CH_3_O-Ph	83.16 ± 1.18	64
**8**	2-F-Ph	67.92 ± 3.37	64
**9**	3-F-Ph	69.00 ± 0.52	64
**10**	4-F-Ph	86.33 ± 1.32	64
**11**	2-Cl-Ph	55.91 ± 8.64	64
**12**	3-Cl-Ph	81.11 ± 2.71	64
**13**	4-Cl-Ph	18.52 ± 10.73	64
**14**	2-Br-Ph	64.67 ± 1.21	64
**15**	3-Br-Ph	62.24 ± 2.05	32
**16**	4-Br-Ph	100.55 ± 0.30	16
**17**	2-CF_3_-Ph	62.23 ± 0.66	64
**18**	3-CF_3_-Ph	65.30 ± 3.55	64
**19**	4-CF_3_-Ph	98.68 ± 0.19	16
**20**	2-NO_2_-Ph	49.26 ± 2.05	64
**21**	3-NO_2_-Ph	96.34 ± 0.59	32
**22**	4-NO_2_-Ph	87.10 ± 0.32	64
**23**	2,4-diNO_2_-Ph	95.70 ± 1.07	4
**24**	pyridin	96.48 ± 0.54	32
**25**	quinolin	97.95 ± 0.41	8
**26**	naphthalen	40.43 ± 3.12	64
**27**	6-CH_3_O-naphthalen	54.48 ± 1.03	>64
**28**	thiophen	82.17 ± 1.69	64
**29**	furan	71.53 ± 5.90	64
**30**	3,4,5-triCH_3_O-Ph	74.98 ± 1.27	64

^a^ Data shown as mean ± SD of three independent experiments.

**Table 2:** MIC and MBC value of compound **23** against *H. pylori*.

Compound	MIC (μM) ^a^	MBC (μM)	MBC/MIC
Metronidazole	8	32	4
**1**–**23**	4	8	2

^a^ Data shown in Table 1.

## Data Availability

Data are also reported in the manuscript.
